# Preliminary investigation of a polyethylene glycol hydrogel "nerve glue"

**DOI:** 10.1186/1749-7221-4-16

**Published:** 2009-09-15

**Authors:** Jonathan Isaacs, Ivette Klumb, Candice McDaniel

**Affiliations:** 1 Department of Orthopaedic Surgery, Virginia Commonwealth University Health System, Richmond, Virginia, USA; 2 Division of Plastic and Reconstructive Surgery, Department of General Surgery Virginia Commonwealth University Health System, Richmond, Virginia, USA

## Abstract

**Background:**

Polyethylene glycol (PEG) hydrogel is a biocompatible semi-adherent gel like substance that can potentially augment nerve repair much like a fibrin sealant. Potential advantages of this substance include fast preparation and set up time, as well as adhesion inhibiting properties. The purpose of this study was to perform an initial evaluation of PEG hydrogel in this application.

**Methods:**

The sciatic nerves of 29 rats were transected and repaired using two 10-0 nylon sutures and either PEG hydrogel or fibrin glue. After 10 weeks, contraction forces of the reinnervated muscles were evaluated and histological assessment of scar tissue performed.

**Results:**

Muscle strength testing revealed the average ratio of experimental to control sides for the fibrin glue group was 0.75 and for the PEG hydrogel group was 0.72 (no significant difference). Longitudinal sections through the nerve repair site showed no significant difference in nerve diameter but did demonstrate a significant reduction in scar thickness in the PEG hydrogel group (p < 0.01).

**Conclusion:**

Though further study is necessary to fully evaluate, PEG hydrogel results in less scar tissue formation and equivalent muscle recovery as fibrin sealant when applied as a nerve glue in a rodent sciatic nerve repair model.

## Background

The use of "surgical glues" to facilitate efficient nerve repairs is an appealing and popular concept. Autologous and commercially available fibrin sealants, such as Tisseel (Baxter Healthcare Corporation, Westlake Village, CA), are the most commonly used substances for this application. While this usage is supported by clinical and laboratory data [1-8], there are concerns regarding ultimate repair strength as well as scar generation [8-11]. Because of these concerns, we began investigating alternative "surgical glues" including polyethylene glycol (PEG) hydrogel (marketed as DuraSeal, Confluent Surgical, Inc., Waltham, MA).

Like fibrin glues, DuraSeal is applied as two separate components: one is a water-soluble amine solution and the other is a multiarmed polyethylene glycol based solution. As these combine, cross-linking results in the rapid formation of a strong adherent gel like substance[12] which can be applied as a cocoon or cylinder around approximated nerve ends. Previously published biomechanical data demonstrated that the holding strength of DuraSeal when applied in this manner is equivalent to commercially available fibrin glues[13]. DuraSeal has proven to be safe and nontoxic when applied as a dural sealant following cranial neurosurgery[12]. Possible advantages of using this or a similar PEG hydrogel as a "nerve glue" include delayed breakdown when compared with fibrin glues and possible adhesion inhibiting properties[14,15] that could prevent peri-neural scarring. The purpose of this study is to investigate this potential role of a polyethylene glycol hydrogel by directly comparing it to a commercially available fibrin glue in a rat sciatic nerve repair model.

## Methods

**Figure 1:**
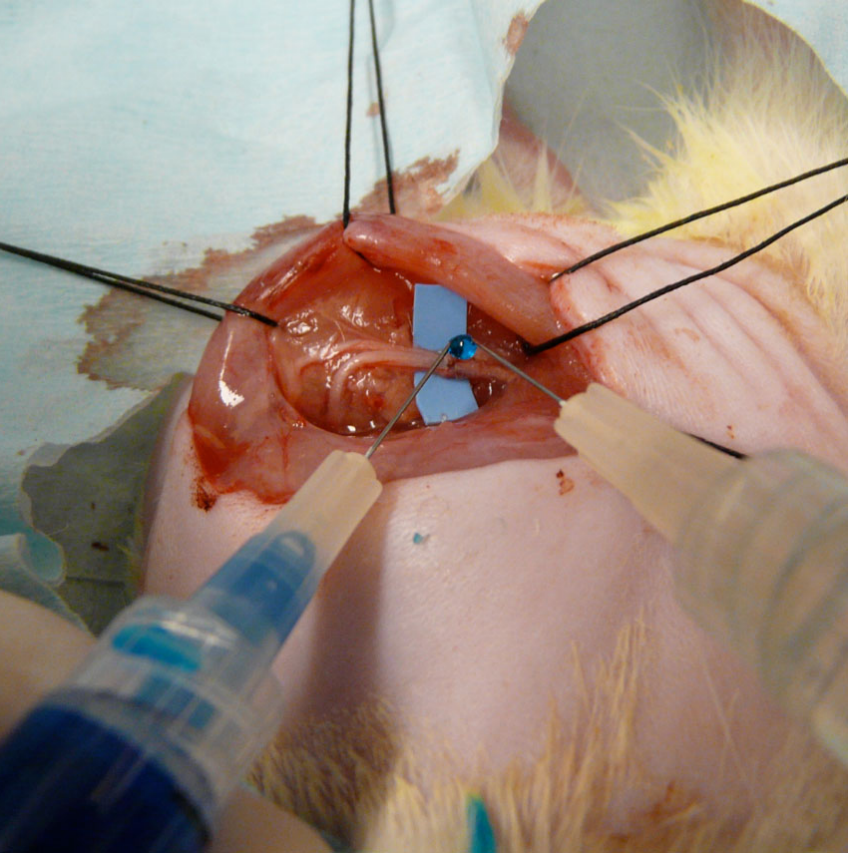
**Application of PEG hydrogel: both components (a water-soluble amine solution and a multiarmed polyethylene glycol based solution) are mixed as small drops formed at the tip of separate needles are touched, forming a suspended drop;**.

The left lower extremity of 30 immature female Sprague-Dawley rats (200 gm) were shaved, prepped with beta-dine, and draped with sterile towels after induction of general anesthesia. Anesthesia was induced and maintained using 2-5% isoflurane gas continuously administered via a nose cone throughout the procedure. The sciatic nerve was exposed (semi-tendinosis biceps femoris splitting approach), isolated, and transected midway between the spine and the knee. Under operating microscope magnification, the cut ends were co-apted with two 10-0 nylon epineural sutures placed 180 degrees apart. A small piece of rubber background (5 × 10 mm) was placed behind the repair site to assist in glue application. Half of the repairs were "glued" with Tisseel and the remainder with DuraSeal (Figure 1). Both products were prepared as described in the manufacturer's insert. The fibrin glue (or PEG hydrogel) was applied circumferentially around the approximated nerve ends. The piece of rubber background was wrapped around the congealing mixture to encourage the formation of a cylinder of glue. This was held for 3 minutes before carefully pealing away the piece of rubber while taking care to leave the cylinder in place. The wound was closed with 4-0 nonabsorbable monofilament and the animals were allowed to recover from anesthesia before being returned to their cages. They were maintained on water and rat chow and monitored daily for signs of illness or distress.

At 10 weeks post surgery, the rats underwent a second surgery in which muscle function or contraction strength of both lower limbs was tested. After the induction of general anesthesia, both the right and left sciatic nerves were exposed as before. The achilles tendon was exposed. The gastrocnemious muscle was isolated and its portion of the achilles tendon secured to a 4-0 silk suture. The leg being tested was fixed to a testing table with intra-osseous pins through the femoral condyles and the distal tibia. The suture through the achilles was coupled to a force transducer (ADInstruments, Inc., Colorado Springs, CO). A supra maximal stimulus of 5 V was applied to the proximal sciatic nerve for 25 ms. The force of the gastrocnemious contraction was measured with the force transducer and recorded using the PowerLab data acquisition system (ADInstruments, Inc., Colorado Springs, CO) and the Apple iBook lap top computer (Apple Computer, Inc., Cupertino, CA). The data was analyzed as a percentage of the experimental to control sides and paired student t-test statistical analysis was performed.

**Figure 2:**
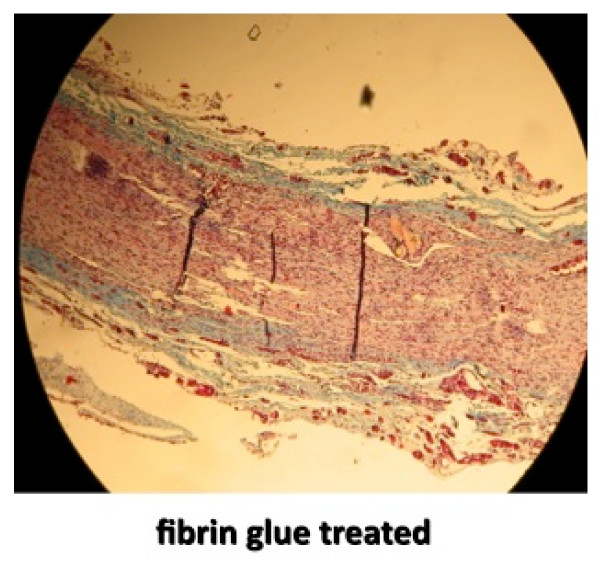
**Longitudinal slide of repair site with fibrin glue, stained with Masson's Trichrome stain for collagen and magnified 10×**.

**Figure 3:**
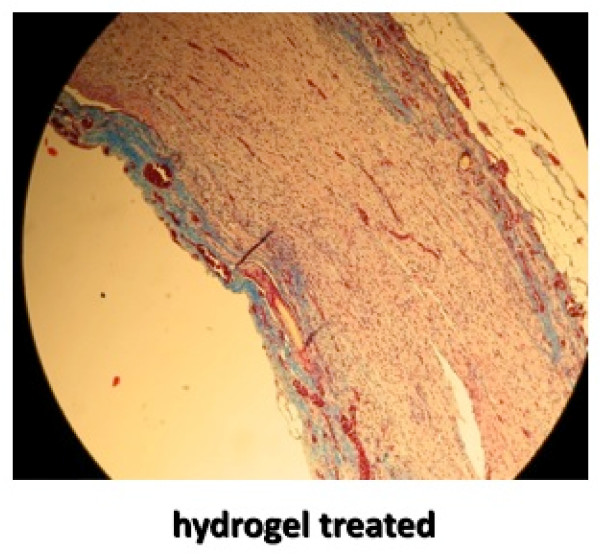
**Longitudinal slide of repair site with PEG hydrogel, stained with Masson's Trichrome stain for collagen and magnified 10×**.

The final analysis of the repaired nerve was by histologic inspection to assess scar formation. The nerves were harvested and fixed in 10% formalin solution. Longitudinal sections were taken at the repair site and stained with Masson's Trichrome. The 10× images were digitalized and peri-neural scarring was evaluated (Figures 2 and 3). A ratio between the thickness of the scar and the nerve diameter was calculated using longitudinal sections through the middle plane of the nerve at the level of the repair. Final statistical analysis was performed between the two groups using paired student t-test.

## Results

**Figure 4:**
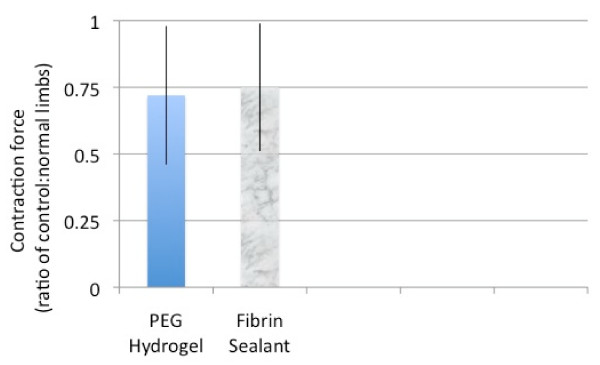
**Comparison of contraction strength ratios (glued side: normal side) for the fibrin sealant and PEG hydrogel groups**.

Twenty-nine of the original 30 rats survived to complete the study. Fifteen were in the hydrogel group and fourteen in the fibrin glue group. Maximal medial gastrocnemious contractile force was measured in all 29 rats. The amplitude of the force transducer waveform was recorded, and a ratio between the experimental and uninstrumented sides for each animal was obtained. Muscle strength testing revealed the average ratio of experimental to control sides for the fibrin sealant group was 0.75 (+/- 0.24)and for the hydrogel group was 0.72 (+/- 0.26). No significant differences (using paired Student's t-test) were demonstrated between these groups. (Figure 4)

Peri-neural scarring was evaluated histologically at 10 weeks in all 29 rats. A ratio between the thickness of the scar (collagen stained with Masson's Trichrome) and the nerve diameter was calculated using longitudinal sections through the middle plane of the nerve at the level of the repair. These measurements demonstrated no significant difference in nerve diameter between the two groups. However, there was a significant reduction in scar thickness in the hydrogel group (P < 0.01, Student's t-test).

## Discussion

The use of a substance to "glue" nerve endings together is appealing for several reasons. Microsurgical suture neurorrhaphy, currently the gold standard, is technically demanding, time consuming, and traumatizes the nerve ends. Therefore, repairing nerves without or at least with fewer sutures should be theoretically easier, faster, and, if truly less traumatizing, then better. Substances currently being used in this capacity have partially achieved this potential. Autologous blood clot was first applied to approximated nerve ends in the 1940's[16] but the use of commercially available fibrin glues, such as Tisseel, has emerged as the most common nonsuture primary or augmenting method of nerve repair. This observation is supported by Tisseel gluing techniques being regularly included in nerve textbooks and international instructional courses [17-19]. Its effectiveness and ease has been documented in several animal nerve repair models [3-8] and clinical outcomes reports[1,2]. Concerns, however, have persisted regarding ultimate repair strengths and scar tissue proclivity [9-11]. Additionally, in this author's opinion, preparation, application, and set up of Tisseel can be cumbersome and, still, relatively time consuming. Because of concerns about repair strength, a few sutures are often still used in the repair, which partially negates advantages of technical ease and decreased nerve trauma.

Polyethylene glycol hydrogel, marketed as DuraSeal, was brought to our attention as a possible nerve glue by a neurosurgeon colleague currently using the product in its FDA approved capacity (and as the name implies) as a dural sealant. DuraSeal sets up in about 2 seconds and is easy to apply as a consistent adhesive cocoon around approximated nerve ends. Clinical and laboratory data has already demonstrated a lack of neurotoxicity[15,20]. Since, holding strength is a key prerequisite for any "glue", this was our initial focus. In a biomechanical comparison of the effects of augmenting a two suture epineural repair of a median sized nerve, DuraSeal demonstrated equivalent holding strength to Tisseel, though it should be noted that while both substances resisted gapping neither significantly increased the ultimate strength of the repair[13].

These results were encouraging enough to prompt further investigation with a direct in vivo comparison between DuraSeal and Tisseel. For the purposes of this initial study, a standard rodent sciatic nerve repair model focusing on functional motor recovery and scar tissue formation was utilized. Despite the well-known disadvantages associated with the superior regenerative powers of the rodent, (which should be kept in mind while interpreting the data), similar rodent models are routinely used in similar preliminary investigations[4,9-11,21-25]. The repair was performed with two epineural sutures before applying either nerve glue which reflects the senior author's current clinical practice. PEG Hydrogel, like fibrin glue, does not add significant holding strength to the repair but does decrease gapping and is being used here only to augment the suture repair[13]. Likewise, since the goal of this study was to compare a novel "nerve glue" to the gold standard "nerve glue" no comparison to suture only repairs was performed. Multiple studies have compared "glue" and sutures with variable results[4-9,11], leaving us to conclude that both techniques are effective. Muscle contraction was the primary outcome parameter focused on and the recorded force measurements were compared as direct ratios of the experimental limb to the contra-lateral normal "control" limb. This data demonstrated no difference between the fibrin glue and PEG hydrogel groups and indirectly implied that either modality could result in acceptable motor recovery. Mean generated force with a supramaximal stimulation of the sciatic nerve was around 75%, which is similar to other nerve repair techniques[26]. Though nerve conduction studies and axonal counts would have been more informative and helpful in a more extensive comparison, the equivalency of muscle contractions between the two groups suggests at least effective motor axon regeneration in both groups.

Measurement of repair site nerve diameter and collagen thickness was performed to assess scar tissue formation and potential nerve compression as described by similar reports making observations on nerve fibrosis[10,21]. Though the nerves were not stretched, slides were prepared as consistently as possible between the two groups with regards to avoiding undulation and comparing equivalent samples though perfect standardization could not be guaranteed. Our findings that nerve diameter was the same between the two groups suggests that PEG hydrogel swelling, which has been reported[27], was not a problem. Masson's Trichrome staining of collagen, however, did demonstrate significantly more scar tissue around the repair site repaired with fibrin glue. Past experience in exposing peripheral nerve tissue to new surgical adhesives have resulted in some unexpected and catastrophic results prompting our interest in perineural scarring. Both cyanoacrylate glue[21] and BioGlue (CryoLife, Inc., Kennesaw, GA) (bovine albumin and glutaraldehyde)[28] have been associated with neurotoxicity and marked fibrotic response around exposed nerves. Additionally, one criticism of the use of fibrin glues in general is that they may generate excessive scar tissue. Herter found that multiple components found in fibrin glue all actually induced fibrosis[10]. PEG hydrogel, on the other hand, has been shown to inhibit adhesion formation in a rabbit pericardial abrasion model[29], a canine durotomy repair model[15], and a porcine intra-abdominal adhesion model[14]. This potential advantage of PEG hydrogel "nerve glue" did not translate to superior results in our rodent model.

## Conclusion

Our preliminary comparison of fibrin sealant with Polyethylene Glycol hydrogel when applied as an augmenting nerve glue in a rodent sciatic nerve repair model suggests an equivalency in motor recovery. Though less scar tissue formation was associated with PEG hydrogel the significance of this is not known. A more extensive comparison of the two substances may be necessary before any definitive conclusion of superiority can be drawn.

## Competing interests

The authors declare that they have no competing interests.

## Authors' contributions

JI was responsible for conception and design, was intimately involved in experimental surgeries, data collection, interpretation of data, and writing the manuscript.

IK performed significant portions of experimental surgeries, data collection, histological preparation and interpretation, and was involved in writing of the manuscript.

CM performed significant portions of data collection.
